# Age, growth and maturity of the Australian sharpnose shark *Rhizoprionodon taylori* from the Gulf of Papua

**DOI:** 10.1371/journal.pone.0206581

**Published:** 2018-10-31

**Authors:** Leontine Baje, Jonathan J. Smart, Andrew Chin, William T. White, Colin A. Simpfendorfer

**Affiliations:** 1 National Fisheries Authority, National Capital District, Port Moresby, Papua New Guinea; 2 Centre for Sustainable Tropical Fisheries and Aquaculture & College of Science and Engineering, James Cook University, Townsville, Queensland, Australia; 3 SARDI Aquatic Sciences, Adelaide, South Australia; 4 CSIRO Oceans & Atmosphere, Hobart, Tasmania, Australia; 5 CSIRO Australian National Fish Collection, National Research Collections Australia, Hobart, Tasmania, Australia; Hellenic Centre for Marine Research, GREECE

## Abstract

Coastal sharks with small body sizes may be among the most productive species of chondrichthyans. The Australian sharpnose shark (*Rhizoprionodon taylori*) is one of the most productive members of this group based on work in northern and eastern Australia. However, life history information throughout the remainder of its range is lacking. To address this knowledge gap, the age, growth and maturity of *R*. *taylori* caught in the Gulf of Papua prawn trawl fishery in Papua New Guinea, were studied. One hundred and eighty six individuals, comprising 131 females (31–66 cm TL) and 55 males (31–53 cm TL) were aged using vertebral analysis and growth was modelled using a multi-model approach. The lack of small individuals close to the size at birth made fitting of growth curves more difficult, two methods (fixed length at birth and additional zero aged individuals) accounting for this were trialled. The von Bertalanffy growth model provided the best fit to the data when used with a fixed length-at-birth (L_0_ = 26 cm TL). Males (*L*_∞_ = 46 cm TL, *k* = 3.69 yr^-1^, L_50_ = 41.7 cm TL and A_50_ = 0.5 years) grew at a faster rate and matured at smaller sizes and younger ages than females (*L*_∞_ = 58 cm TL, *k* = 1.98 yr^-1^, L_5o_ = 47.0 cm TL and A_50_ = 0.93 years). However, none of the methods to account for the lack of small individuals fully accounted for this phenomenon, and hence the results remain uncertain. Despite this, the results reaffirm the rapid growth of this species and suggest that the Gulf of Papua population may grow at a faster rate than Australian populations. *Rhizoprionodon taylori* is possibly well placed to withstand current fishing pressure despite being a common bycatch species in the Gulf of Papua prawn trawl fishery. However, further research needs to be undertaken to estimate other key life history parameters to fully assess the population status of this exploited shark species and its vulnerability to fishing in the Gulf of Papua.

## Introduction

A general view on the life history characteristics of sharks assumes slow growth, late maturity, and a low number of offspring resulting in populations that have low intrinsic rates of population growth and are highly vulnerable to overfishing [1, 2]. However, not all shark species share these characteristics. In particular, small-bodied carcharhinids such as the milk shark *Rhizoprionodon acutus* and the sliteye shark *Loxodon macrorhinus* are characterised by relatively rapid growth and early maturity resulting in higher population turnover rates [3, 4]. Fast population turnover rates for these species make them potentially more resilient to fishing [5], although sustainable shark catch is mostly associated with the development of science-based fisheries management in countries [6].

The Australian sharpnose shark *Rhizoprionodon taylori* is a small carcharhinid species known to have one of the fastest growth rates of all shark species [7, 8]. Initial studies suggested it grows rapidly in the first year of life, on average increasing to 140% of its length-at-birth, and attains a maximum length of only 67 and 97 cm TL respectively in different locations in Australia [8, 9]. Maturity is reached after only one year with a litter of 1–10 pups produced every year following maturity [8, 10]. *Rhizoprionodon taylori* is also one of the few elasmobranch species that can halt embryonic development (diapause), possibly to facilitate increased litter sizes [10, 11]. Occurring only in southern New Guinea and tropical and sub-tropical nearshore waters of Australia from Carnarvon in Western Australia to Moreton Bay in southern Queensland, it is a locally abundant species often incidentally caught in trawl and gillnet fisheries [12, 13].

All known biological information about *R*. *taylori* has been established from populations in Australia [8–10, 14–16]. Recent trawl fisheries data from Papua New Guinea (PNG) confirm that *R*. *taylori* is also frequently caught as bycatch in the Gulf of Papua (GOP) (NFA unpublished data). Prawn trawling has occurred in the area since the late 1960’s and bycatch levels can comprise up to 85% of the total catch [17]. However, the effect of trawling on the sustainability of bycatch populations cannot be properly assessed without determining species compositions and locally relevant biological parameters.

Life history traits can differ for populations in separate localities [18, 19]. The Gulf of Papua (GOP) is in close proximity to the northern coast of Australia. However, *R*. *taylori* has been observed to maintain residency in embayments and nearshore habitats, travelling short distances and rarely moving greater than 100 km within 6 months to one year [20]. These limited movements mean that there may be differences in the life history of this species between the GOP and other regions. These differences need to be investigated since variations in size at birth and length-at-maturity could affect fisheries risk assessments, and have already been documented between different locations in Australia [9, 10, 15].

Age and growth studies provide essential information for wider population analyses such as stock assessments [21]. Growth parameters for *R*. *taylori* were determined by [8] prior to the development and use of multiple growth models within an information theoretic framework, which is now the recommended approach for age and growth studies [5, 22]. This study used the more contemporary multi-model approach to determine growth and maturity parameters for *R*. *taylori* in the GOP. The specific aims were: (1) to determine the age, growth and maturity of *R*. *taylori*; (2) compare life history parameters to previous work to determine if the use of the multiple model approach substantially changed the outcomes; and (3) examine spatial variation in life history of this species. This study also contributes new knowledge from a data poor region that can be used to inform fisheries management and conservation in PNG.

## Materials and methods

### Sample collection

This work is a collaboration with the National Fisheries Authority (NFA), the government agency responsible for managing commercial fisheries and implementing fisheries research in PNG. Fishery observers were stationed on board prawn trawlers and collected sharks that were caught as bycatch and discarded. The sharks collected for this study had already suffered mortality in the process of fishing and no sharks were intentionally sacrificed for the study. All sampling procedures were allowed by the NFA and in line with James Cook University, Animal Ethics approval A2310 obtained prior to the commencement of the study. Sampling did not involve endangered or protected species. No further permits were required by relevant authorities.

**Fig 1:**
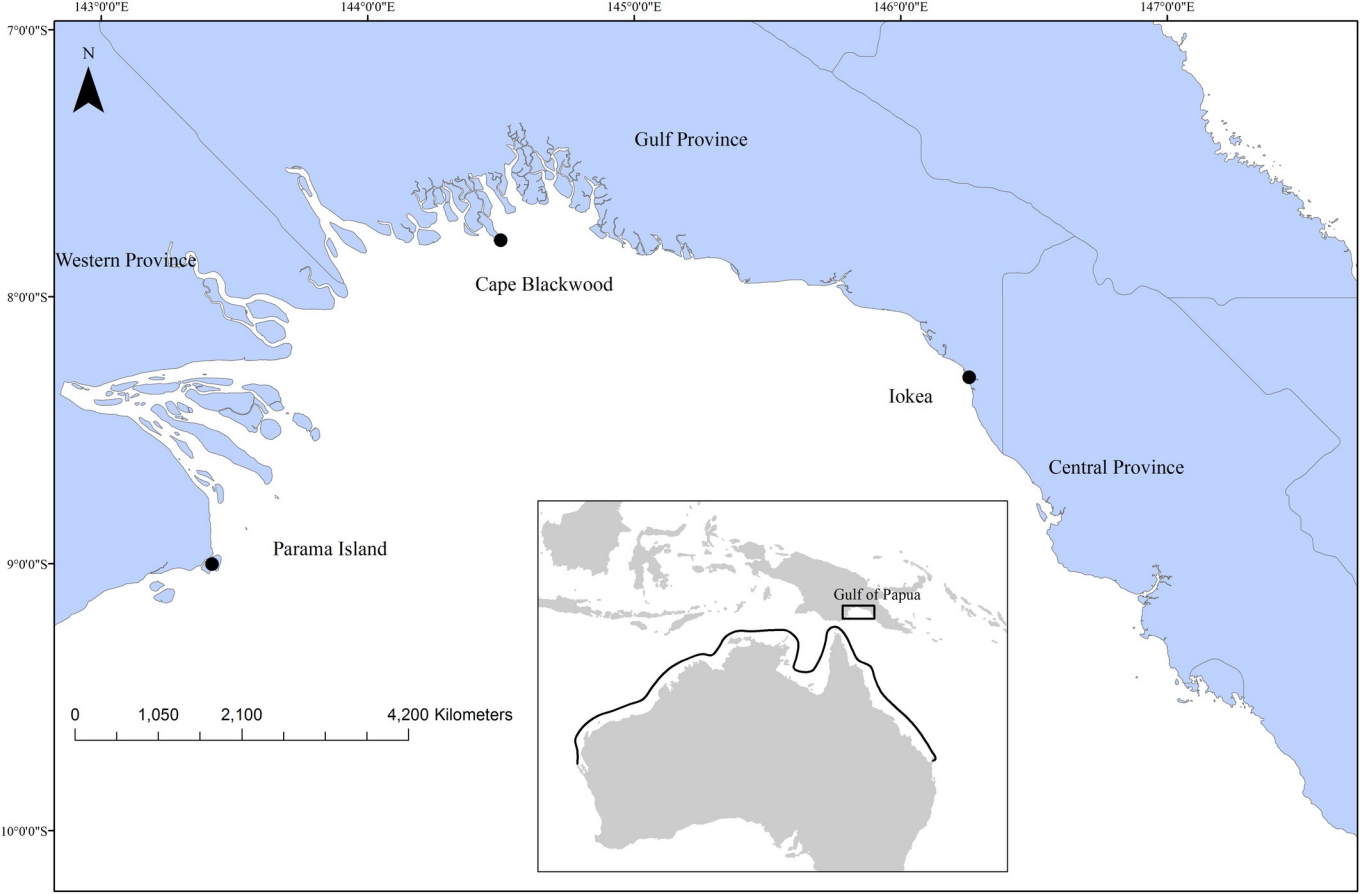
The Gulf of Papua is situated along the southern coast of Papua New Guinea. The insert shows the distribution of *Rhizoprionodon taylori* in Australia.

Commercial trawling in the GOP occurs between Parama Island in the West, just south of the mouth of the Fly River, and the border of the Central and Gulf Provinces in the East (Fig 1). Trawl fishing is permitted all year round throughout the GOP except in a section of the Gulf between Iokea and Cape Blackwood which is closed to fishing between the 1st of December and the 31st of March, a measure put in place to protect the growth and survival of prawn recruits [23]. Samples of *R*. *taylori* were collected on commercial vessels from June 2014 to August 2015. Whole samples were kept frozen and brought ashore at the end of each trip for confirmation of identification and processing. In a laboratory samples were defrosted, total length (TL) measured, and sex recorded. For each individual, maturity was also determined using an index modified from [24]. Reproductive organs were examined and categorised according to the developmental stage of the ovaries and uteri in females, and claspers in males. Females were categorised into one of five stages and males into one of three stages (Table 1). A section of the vertebral column from beneath the first dorsal fin was retained and stored frozen for subsequent age determination [5, 25].

**Table 1:** The maturity of male and female samples were determined by the state of the uteri and ovaries in females, and claspers in males. Maturity stages were assigned a binary category for statistical analysis.

Female stage		Description	Binary category
**1**	Immature	Uteri very thin, ovaries small and without yolked eggs.	0
**2**	Maturing	Uteri slightly becoming enlarged at one end, ovaries becoming larger and small yolked eggs developing.	0
**3**	Mature	Uteri large along entire length, ovaries containing some large yolked eggs.	1
**4**	Pregnant	Uteri containing embryos or large eggs.	1
**5**	Post-partum	Uteri very large but without embryos.	1
**Male stage**			
**NC**	Not Calcified	Clasper very short not extending past the pelvic fin tip.	0
**PC**	Partially Calcified	Claspers longer, extending past the pelvic fin tip, not entirely hard, still flexible.	0
**FC**	Fully Calcified	Claspers long, hard along almost the entire length.	1

### Vertebrae preparation

Vertebrae processing and aging followed protocols described by [26]. Frozen vertebrae were thawed and any excess tissue was removed using a scalpel. Vertebrae were separated into individual centra and immersed in 4% sodium hypochlorite solution for 3–5 minutes to clean remaining soft tissue from the small sized vertebrae. The centra were then rinsed using water and dried in an oven at 60°C for 24 hours. A single centrum was selected from each individual and mounted on a microscope slide using Crystal bond adhesive (SPI supplies, Pennsylvania, USA). To achieve the desired thickness of < 400 μm the vertebrae was sanded towards the centre of the centrum using 400–1200 grit wet and dry abrasive paper. After one side was complete the centrum was remounted and sanded again on the other side until the desired thickness was achieved [8].

### Age determination

To estimate the age of each individual, mounted sections of vertebrae were observed using a dissecting microscope. Growth increments appeared as a pair of alternating wide opaque band and a narrow translucent band, referred to as a band pair [5, 26]. The birthmark was identified where there was an obvious change in angle along the corpus calcareum. Subsequent band pairs that spanned from one side of the corpus calcareum to the other side were interpreted to represent annual growth [5, 25]. The age of each individual was estimated as the number of band pairs present after the birthmark. The annual deposition of bands for this species has been validated using marginal increment analysis and size frequency data by [8].

### Precision and bias

Visual estimation of age from vertebrae is an approach which may include some level of bias [25]. To minimise bias two readers estimated ages separately. The first reader conducted an initial read of all vertebrae followed by a second experienced reader. Both readers had no prior knowledge of the sex or size of individuals. Final ages were the result of a consensus process between the readers–where counts were different readers examined the section and agreed on a final age. Where differences could not be resolved, those centra were removed from the analyses. To assess the precision of counts the average percent error (APE) [27], Chang’s coefficient of variation (CV) [28] and percent agreement (PA ± 1 year) [25] were used. Bowker’s test of symmetry was used to estimate bias between readers [29]. Analyses were carried out using ‘FSA’ package version 0.8.11 in the R program environment version 3.2.2 [30].

### Partial ages

For a species that reproduces seasonally, and the period of parturition is known, it is possible to assign partial ages and therefore improve age estimation [31]. The pupping season for *R*. *taylori* was observed in January in Queensland [8]. In this study the largest embryo (22 cm TL) was caught in the month of December, confirming a similar timing in the GOP. Partial ages were calculated by choosing a birth date of 15th of January and determining the total number of days between this date and the date of capture which was then divided by the number of days in a year. This value was added to the number of full annual band pairs for each individual to give the final age. For example, samples aged at 1 year caught on the 17th of June and 30th of August, respectively, were given partial ages of 1.39 and 1.62 years.

### Growth model fitting

The growth of *R*. *taylori* was modelled using a multi-model approach. This method incorporated the Akaike Information Criterion (AIC) [32] which selected the best model fit based on the lowest AIC value [33]. Preference for the use of multiple growth models over an *a priori* approach, using only the von Bertalanffy growth model (VBGM) is standard methodology in elasmobranch growth literature [22]. The multi-model approach is considered to provide better growth estimates as it avoids model mis-specification and biases compared to the use of a single model [22, 26, 34]. The lack of small juveniles in the sample, and their likely very rapid growth required a variety of approaches to determine the most suitable growth parameters. Three candidate models were used: VBGM, logistic model, and Gompertz model (Table 2). However, because of the limited data from very young individuals three approaches to fitting the models was used: (1) standard three-parameter growth models, (2) versions of the growth models with a fixed length-at-birth (which ensured that models accounted for the rapid early growth; two-parameter version) [35], and (3) three-parameter models with four hypothetical aged zero individuals (L_0_ = 26 cm TL) added to the sample in order to provide a reference point for the model given that aged zero individuals were absent from the sample [31]. Separate growth models were constructed for males, females, and combined sexes.

**Table 2:** Equations of the three growth functions used in the multi model approach.

Model	Growth function
von Bertalanffy	(*t*) = *L*_0_ + (*L*_∞_ − *L*_0_)(1 − *e*^(−*kt*)^)
Logistic	$L(t)=\frac{L_{\infty }L_{0}(g_{\left(log\right)}t)}{L_{\infty }+(L_{0}\;e^{{(g}_{\left(log\right)}t-1)})}$
Gompertz	$L(t)=L_{\infty }\;{e(}^{-L_{0}\;e^{\left(-g_{\left(gom\right)}t\right)}})$

The three-parameter models estimated length-at-birth (*L*_*0*_), asymptotic length (*L*_*∞*_) and the different growth coefficients for each respective model; *k* indicates the relative growth rate of the VBGM model while *g*_(*log*)_ and *g*_(*gom*)_ represent alternative sigmoidal growth of the Gompertz and logistic models [36]. The two-parameter models incorporated a fixed known value for length-at-birth and thus the models only estimated the asymptotic length and the growth coefficients. Umbilical scars were not recorded in this study which meant that a length-at-birth for *R*. *taylori* in the GOP was not identified, but could be estimated using other data available from the sample as well as published information. In this study the smallest free swimming individuals were 31 cm (TL) and largest embryos were 22 cm (TL) observed in December (a month prior to pupping). The literature estimates of length-at-birth are 25–30 cm [15] from northern Australia and 22–26 cm in north eastern Australia [8]. A possible estimate for the length-at-birth would therefore be 22–30 cm, however in the GOP *R*. *taylori* are still embryos at 22 cm and are possibly born at a larger size. The midpoint between 22 and 30 cm (26 cm) was chosen because this value was within the length-at-birth range suggested by both previous studies and was biologically plausible given embryo sizes in the GOP. Growth models were fit using the ‘nls’ function, multi-model analysis was conducted using the ‘MuMIn’ package version 1.15.6 [37] and bootstrapped confidence intervals were produced using the ‘nlstools’ package version 1.0–2 [38] in the R program environment version 3.2.2 [30].

As the sample size was less than 200, the AIC_C_, a size adjusted bias correction, was used [39]:

$${AIC}_{C}=AIC+\frac{2k(k+1)}{n-k-1}$$

where *AIC* = *nlog*(*σ*^2^) + 2*k*, k is the total number of parameters + 1 for variance σ^2^ and *n* is the sample size. The model that has the lowest *AIC*_*C*_ value (*AIC*_min_) was chosen as the best fit for the data. The AIC difference (Δ) was calculated for each model (i = 1–3) and used to rank the remaining models as follows:

$$\Delta_{i}={AIC}_{Ci}-{AIC}_{min}$$

Models were ranked according to the value of Δ. Values from 0–2 were considered to have the strongest support, less support was given to values between 2–10 and the least support for Δ values > 10 [40]. The AIC weights were calculated by the expression:

$$w_{i}=\frac{{e(}^{-\frac{\Delta_{i}}{2}})}{(\sum_{j=1}^{3}{e(}^{\frac{\Delta_{i}}{2}}))}$$

To test if there were differences in the growth curves for males and females, a likelihood ratio test was carried out [41]. This was conducted on the model with the best fit based on the AIC_C_ results for the sexes combined. The method used to carry out the likelihood ratio test was described by [42] and incorporated into the R program environment version 3.2.2 [30] for this analysis.

### Maturity

The maturity stage data was converted to a binary maturity category (immature = 0 or mature = 1) for statistical analyses (Table 1). The length-at-maturity was estimated for both males and females using logistic regression [24]:

$$P{\left(l)=P_{max}\;(1+e^{-\ln\left(19)(\frac{l-l_{50}}{l_{95-l_{50}}}\right)}\right)}^{-1}$$

where P (*l*) is the proportion mature at TL, *l* and *P*_*max*_ is the maximum proportion of mature individuals. The lengths of which 50 and 95% of the population are mature (*l*_50_ and *l*_95_) were estimated using a generalised linear model (GLM) with a binomial error structure and a logit-link function using the ‘psyphy’ package version 0.1–9 [43] and the ‘FSA’ package version 0.0.11 [44] in the R program environment version 3.2.2 [30]. Age-at-maturity was determined by substituting length with age. A_50_ and A_95_ were the ages at which 50 and 95% of the population reached maturity.

## Results

### Age determination

**Fig 2:**
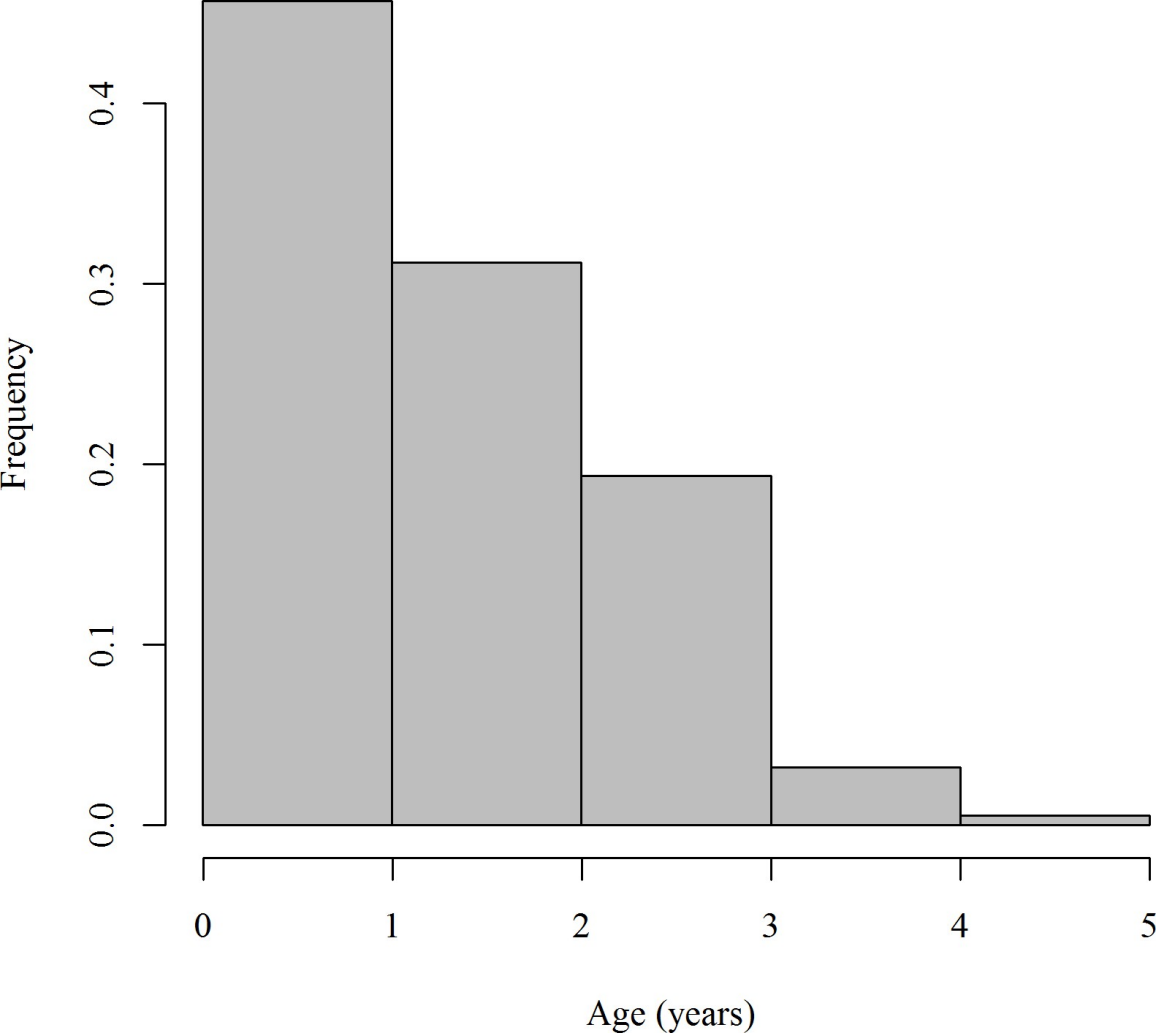
Frequency histogram of samples for each age class.

In total 186 individuals were collected: 131 females and 55 males. Males ranged in size from 31–53 cm (TL) and females from 31–66 cm (TL). The majority of sharks were aged between 0 and 1 years (i.e. birthmark was present but no fully formed 1^st^ band pair) (Fig 2). Final partial ages ranged from 0.2 to 4.6 years. The oldest female was 64 cm (TL) and aged at 4.6 years. The oldest male was 51 cm (TL) and aged at 3.6 years.

**Fig 3:**
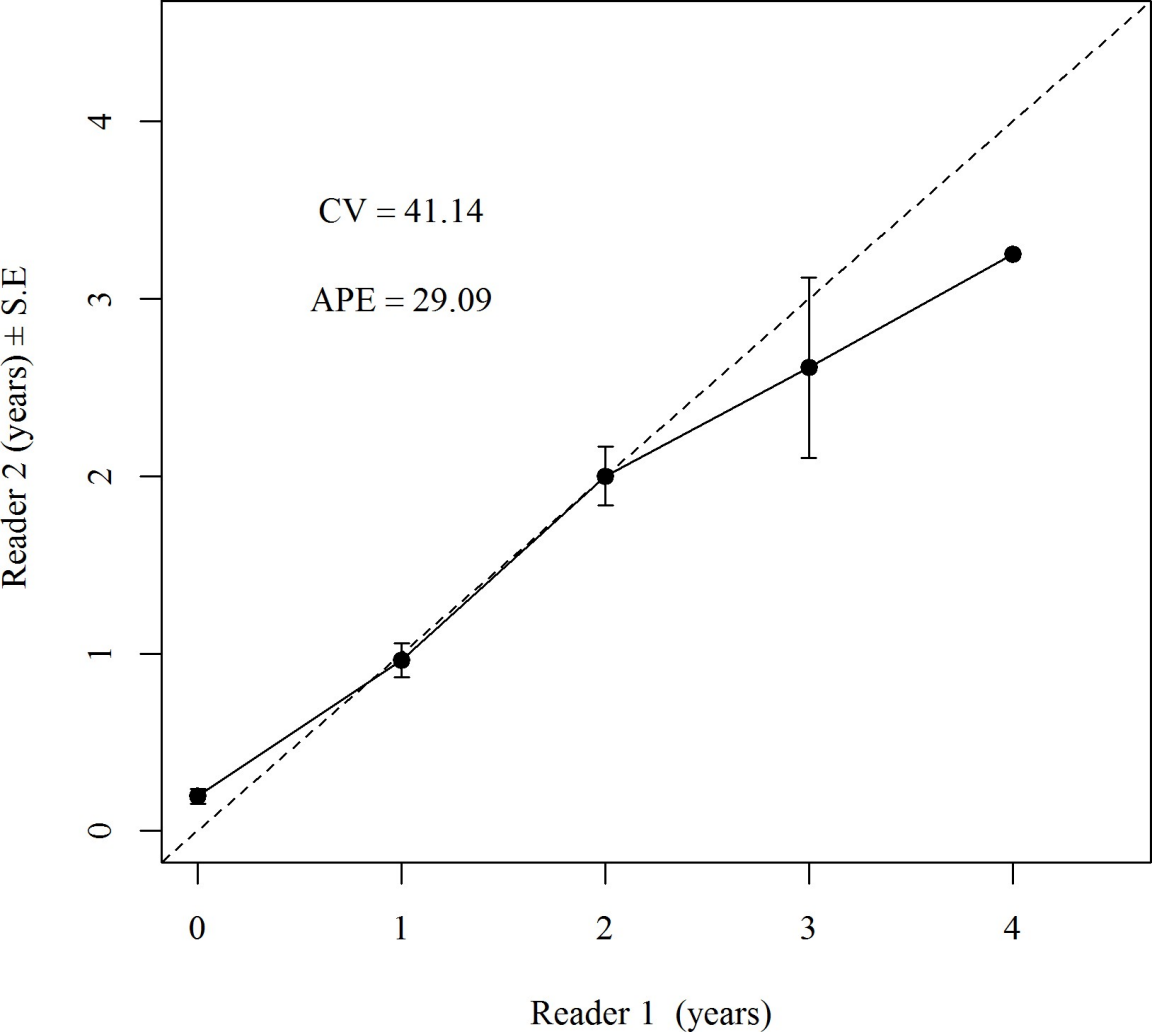
Age bias plot showing agreement between two independent readers. The PA ± 1 year was 62.4%, APE was 29.1 and Chang’s coefficient of variation (CV) was 42.4%.

The measures of variability around the determination of ages were high compared to other elasmobranch aging studies [3, 45, 46]. The Average Percent Error (APE), Chang’s CV and PA ± 1 year were 29.1%, 42.4% and 62.4%, respectively. Higher variability will be experienced when aging short lived species as small differences in band pair counts can produce inflated error estimates in comparison to longer lived species [8]. Bowker’s test for symmetry (df = 8, x^2^ = 16.4, *P* = 0.037) indicated some systematic bias between readers. The age bias plot (Fig 3) showed that this bias was associated with reader 1 estimating younger counts of band pairs at 3 and 4 years relative to reader 2. The use of consensus counts to produce final ages overcame this ageing bias.

### Growth model fitting

**Fig 4:**
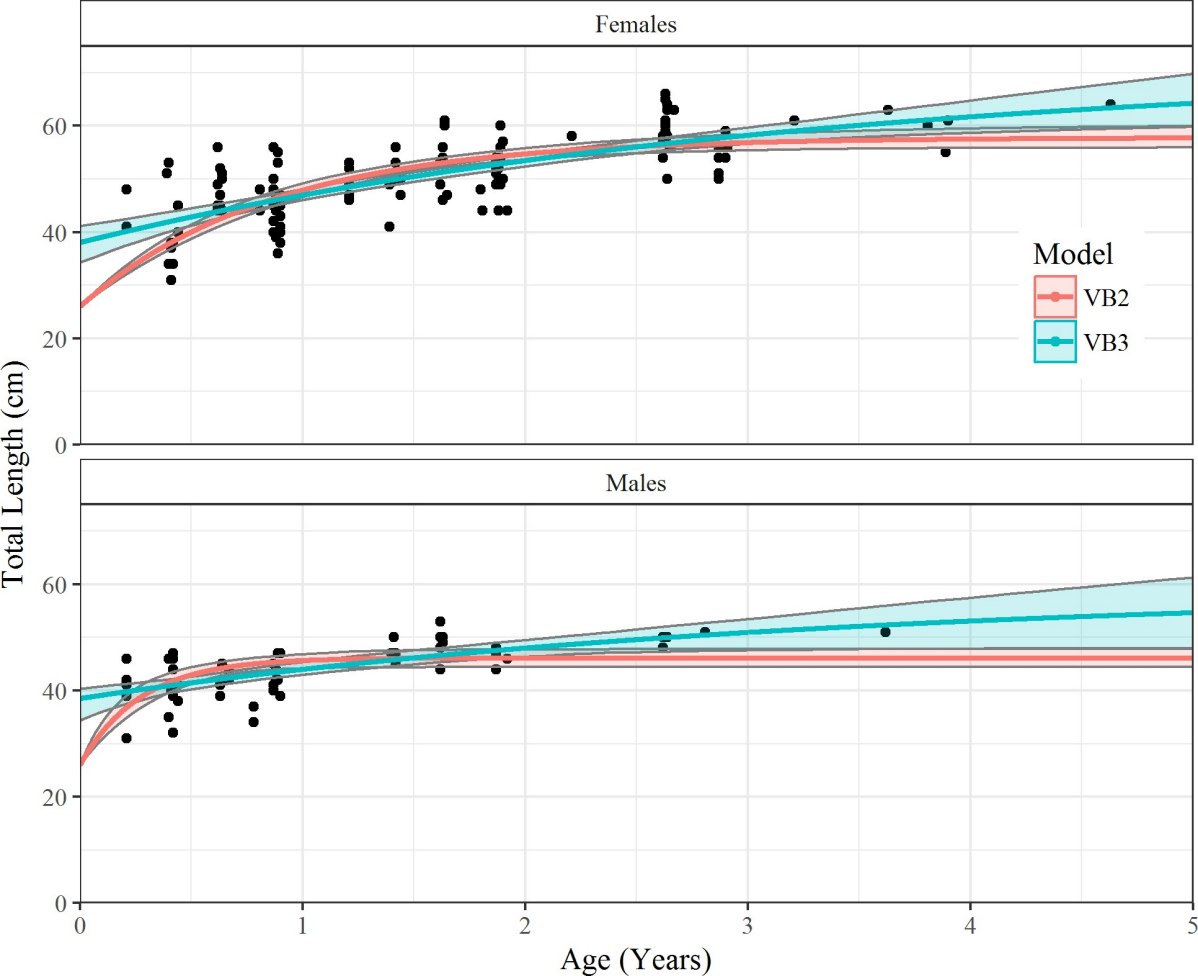
Two (VB2) and three parameter (VB3) length-at-age curves for female and male *Rhizoprionodon taylori* from the Gulf of Papua fitted with 95% bootstrapped confidence intervals.

Without data from small new born animals three-parameter models were unsuitable as the projected length-at-birth values were too high and biologically unreasonable for *R*. *taylori* (37–38 cm) (Table 3). The three-parameter models with the four added size at birth individuals had similar AIC weights for combined and individual sexes (Table 4). All three candidate models had similar weights in the three-parameter models. Neither of the three-parameter approaches accurately represented the early growth of *R*. *taylori*, over-estimating the size at birth. Amongst the two-parameter models the VBGM performed best as neither logistic and Gompertz models had Δ values < 2, although there was some weak support for the Gompertz model for males (*w* = 0.24) (Table 5). The two-parameter models projected much higher growth completion rates (*k*, *g*_(*log*)_, *g*_(*gom*)_) than three-parameter models however, the fixed length-at-birth value were more realistic. Thus, it is likely that none of the fitting approaches produced accurate estimates of all three parameters. However, the two-parameter VBGM is recommended to describe the growth of *R*. *taylori* in the GOP (Fig 4), with a growth estimate (*k*) of 1.27 for both sexes combined (Table 5). A likelihood ratio test showed significant difference (df = 3, x^2^ = 23.3, *P* = 3.5) in the VBGM fit between males and females which demonstrated that sexes should be modelled separately. The error estimates for the male VBGM parameters were much higher than for females, indicating much greater level of uncertainty, probably because of the smaller sample size.

**Table 3:** Summary of results from the multi model approach incorporating Akaike’s information Criterion (AIC) using three-parameter versions of models.

Sex	Model	n	AIC_C_	Δ	W (%)	L_0_(±SE)	L_∞_(±SE)	k(±SE)	g_(log)_(±SE)	g_(gom)_(±SE)	RSE
**Combined**	VB3	186	1129.06	0.53	0.29	37.89±1.27	74.34±12.98	0.25±0.14			4.96
	Logistic	186	1128.53	0	0.38	38.17±1.11	66.92±6.0		0.50±0.14		4.96
	Gompertz	186	1128.78	0.25	0.33	38.03±1.18	69.65±8.21			0.38±0.14	4.96
**Male**	VB3	55	306.3	0.17	0.32	38.48±1.50	58.89±15.72	0.31±0.37			3.72
	Logistic	55	306.13	0	0.35	38.51±0.76	55.71±8.90		0.51±0.20		3.71
	Gompertz	55	306.22	0.09	0.33	38.50±1.44	57.00±11.41			0.41±0.37	3.72
**Female**	VB3	131	801.08	0.29	0.31	38.03±1.90	71.08±10.55	0.31±0.17			5.04
	Logistic	131	800.8	0	0.36	38.53±1.35	66.30±5.79		0.55±0.15		5.04
	Gompertz	131	800.93	0.13	0.33	38.30±1.74	68.17±7.46			0.43±0.17	5.04

n is the sample size, AIC_C_ is the small-sample bias adjusted from the Akaike’s Information Criteria, Δ is the difference in AIC_C_ values between models, w (%) are the AIC_C_ weights, L_0_ and L_∞_ are the length-at-birth and asymptotic length in cm respectively, k is the growth completion rate in (year^-1^) for the VB3, g_(log)_ and g_(gom)_ are the growth parameters for Logistic and Gompertz functions respectively, SE is the standard error of each growth parameter and RSE is the residual standard error for the models.

**Table 4:** Summary of results from the multi model approach incorporating Akaike’s information Criterion (AIC) using three-parameter versions of models with four hypothetical aged zero individuals.

Sex	Model	n	AIC_C_	Δ	W (%)	L_0_(±SE)	L_∞_(±SE)	k(±SE)	g_(log)_(±SE)	g_(gom)_(±SE)	RSE
**Combined**	VB3	190	1166.85	0	0.45	35.12±1.32	63.88±4.03	0.48±0.14			5.15
	Logistic	190	1168.21	1.96	0.23	35.98±1.14	61.75±2.87		0.73±0.15		5.16
	Gompertz	190	1167.59	0.73	0.32	35.59±1.22	62.65±3.33			0.60±0.14	5.16
**Male**	VB3	57	330.66	0	0.39	34.55±1.87	50.42±2.57	1.01±0.43			4.19
	Logistic	57	331.28	0.62	0.28	35.28±0.92	50.41±2.47		1.17±0.25		4.21
	Gompertz	57	331.01	0.35	0.33	34.96±1.76	50.44±2.53			1.08±0.44	4.2
**Female**	VB3	133	819.85	0	0.44	34.91±1.96	63.77±3.92	0.53±0.17			5.17
	Logistic	133	821.06	1.21	0.24	36.22±1.38	62.27±3.04		0.77±0.15		5.20
	Gompertz	133	820.51	0.66	0.32	35.64±1.8	62.92±3.41			0.65±0.18	5.19

n is the sample size, AIC_C_ is the small-sample bias adjusted from the Akaike’s Information Criteria, Δ is the difference in AIC_C_ values between models, w (%) are the AIC_C_ weights, L_0_ and L_∞_ are the length-at-birth and asymptotic length in cm respectively, k is the growth completion rate in (year^-1^) for the VB3, g_(log)_ and g_(gom)_ are the growth parameters for Logistic and Gompertz functions respectively, SE is the standard error of each growth parameter and RSE is the residual standard error for the models.

**Table 5:** Summary of results from the multi model approach incorporating Akaike’s information Criterion (AIC) using two parameter versions of growth models with a fixed length-at-birth for *Rhizopriondon taylori* from the Gulf of Papua.

Sex	Model	n	AIC_C_	Δ	W (%)	L_∞_(±SE)	k(±SE)	g_(log)_(±SE)	g_(gom)_(±SE)	RSE
**Combined**	VB2	186	1193.71	0	0.99	55.95±0.95	1.27±0.11			5.54
	Logistic	186	1213.08	19.38	0	54.41±0.75		2.12±0.14		5.83
	Gompertz	186	1203.61	9.9	0.01	55.07±0.82			1.67±0.13	5.68
**Male**	VB2	55	336.13	0	0.64	46.11±0.9	3.69±0.68			4.44
	Logistic	55	339.47	3.34	0.12	45.08±0.77		6.73±1.23		4.57
	Gompertz	55	338.1	1.97	0.24	45.52±0.82			5.04±0.92	4.52
**Female**	VB2	131	830.37	0	0.96	57.78±1.12	1.17±0.12			5.40
	Logistic	131	842.88	12.52	0.00	56.08±0.84		1.98±0.15		5.66
	Gompertz	131	836.6	6.23	0.04	56.8±0.94			1.55±0.13	5.53

n is the sample size, AIC_C_ is the small-sample bias adjusted from the Akaike’s Information Criteria, Δ is the difference in AIC_C_ values between models, w (%) are the AIC_C_ weights, L_∞_ is the asymptotic length in cm, k is the growth completion rate in (year^-1^) for the VB2, g_(log)_ and g_(gom)_ are the growth parameters for logistic and Gompertz functions respectively, SE is the standard error of each growth parameter and RSE is the residual standard error for the models.

### Maturity

**Fig 5:**
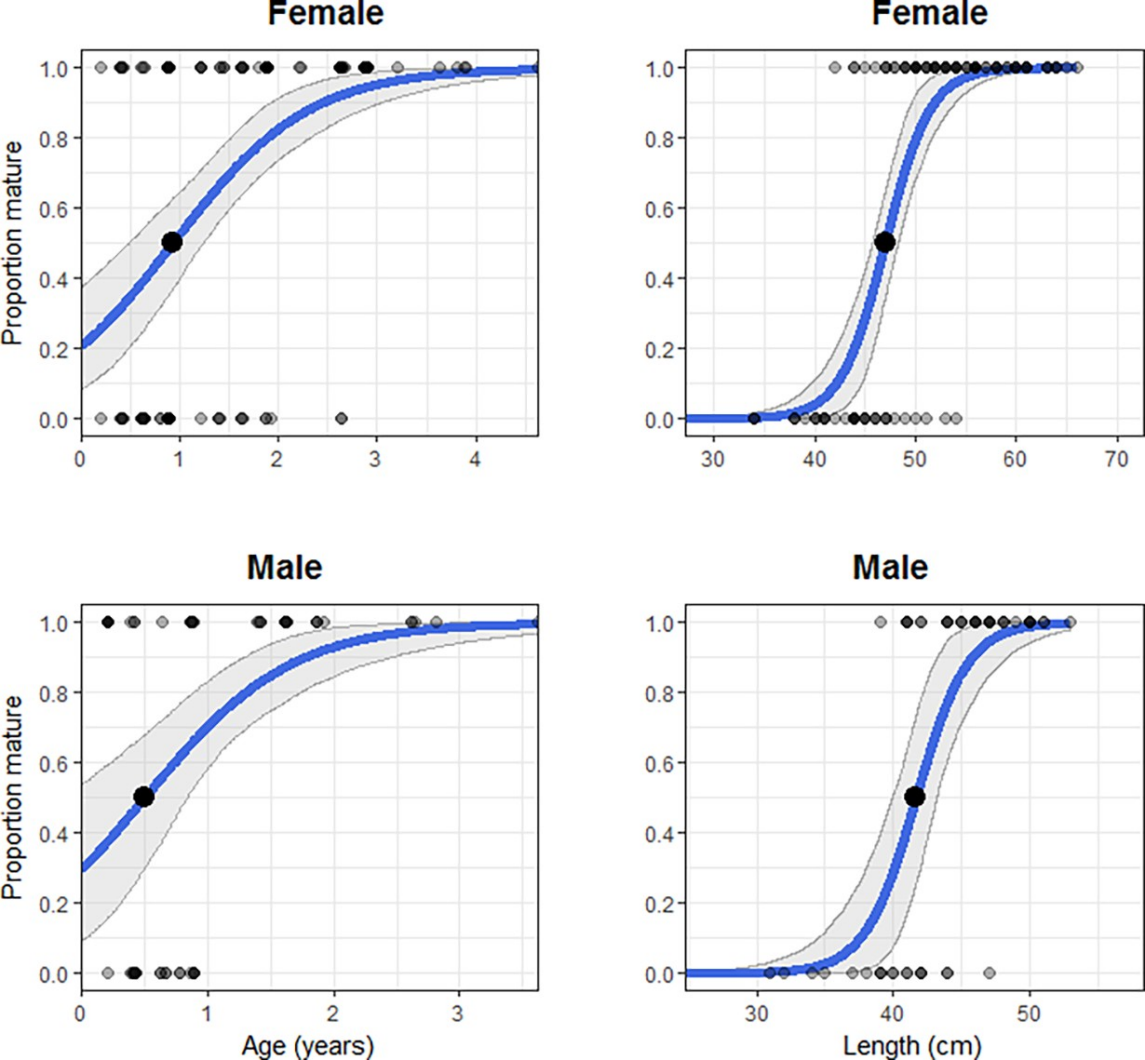
Age and length-at-maturity ogives for female and male *Rhizoporionodon taylori* from the Gulf of Papua. The large points on the curve represent the length and age at which 50% of population reaches maturity. 95% bootstrapped confidence intervals are indicated with shaded areas.

Maturity estimates for male and female *R*. *taylori* differed slightly. Females grew larger than males, and males matured earlier in terms of both length and age (Fig 5). The smallest mature female was 42 cm (TL) and lengths at maturity L_50_ and L_95_ were 47.0 cm (TL) ± 0.68 S.E. and 53.5 cm TL ± 1.2 S.E. The A_50_ and A_95_ were 0.93 years ± 0.1 S.E. and 2.95 years ± 0.4 S.E., respectively for females. The smallest mature male was 39 cm (TL). The L_50_ and L_95_ for males were 41.7 cm (TL) ± 0.8 S.E. and 47.2 cm (TL) ± 1.5 S.E while the ages at maturity A_50_ and A_95_ were 0.5 years ± 0.2 S.E. and 2.2 years ± 0.6 S.E.

## Discussion

The results of this study reaffirm the very rapid growth and maturity of *R*. *taylori* in comparison to the majority of chondrichthyan species. For sharks, von Bertalanffy growth completion rates (*k*) >1, as seen in *R*. *taylori* are rare and indicate very rapid growth compared to other species, most of which reach much larger maximum sizes. For example *Isurus oxyrinchus* (*k* = 0.052 year^-1^) and *Carcharhinus plumbeus* (*k* = 0.040 year^-1^) [47, 48] both have much lower growth completion rates and as a result take many years before they reach maturity. Small bodied coastal shark species such as *Rhizoprionodon terraenovae* (*k* = 0.5 year^-1^) and *Rhizoprionodon acutus* (*k* = 0.63 year^-1^ for females, *k* = 0.94 year^-1^ for males) [4, 49] generally exhibit more rapid growth. *Rhizoprionodon taylori* has the fastest known growth completion rate for a shark species gaining more than 100% of its body size in the first year of life [8].

The growth completion rate of female *R*. *taylori* from the two-parameter model fitting (*k* = 1.165 year^-1^) in the GOP is similar to that previously found in Australia (*k* = 1.013) [8]. The elevated growth completion rate (k = 3.69 y^-1^) for males predicted by the model had a high level of error and so remains to be resolved by further research. The two-parameter VBGM produced reduced L_∞_ estimates for both males and females. While the three parameter VBGMs estimated reasonable values for L_∞_ for both sexes, the L_0_ projected by the model was well beyond the size at birth previously reported for this species and outside the ranges expected from the GOP data. The addition of hypothetical zero aged animals provided little improvement in the value of L_0_ for *R*. *taylori* despite its utility in other species [31]. Given the linkage between the parameters in growth models the true values of growth completion parameters lie between those estimated by the two and three parameter models. Based on the biological implausibility of size at birth projected by the three-parameter VBGM it cannot be considered to model the growth of *R*. *taylori* in the GOP. Similarly the two-parameter VBGM has its drawbacks however does provide growth estimates that are within reason particularly for females, but further investigation is warranted given the large confidence intervals around k for males and the large differences between males and females. Factors that may have influenced this outcome are; methodological differences between this study and [8] who fitted growth curves by eye, the relatively small number of males in the sample and the model being constrained by fixing the length-at-birth. Fixing models by selecting a single length-at-birth value has been discouraged because of variations in the actual birth size [50].

Two-parameter models are recommended under stringent conditions where: there is limited data for smaller juveniles, low sample sizes, and where the length-at-birth cannot be estimated from the study population but can be deduced from a representative population in the same geographic region [34]. The lack of data from younger *R*. *taylori* close to the length-at-birth posed a problem that is usually solved by back calculation [31]. However, this could not be done because much of the growth of *R*. *taylori* occurs prior to the first year of life and there are no growth bands deposited during this period that can be used to track their growth. In addition, although the AIC values indicated that the three-parameter models provided a better fit the projected length-at-birth values that were not biologically realistic. For these reasons, the use of two-parameter models in this study was considered to provide the best way to ensure that biologically plausible parameters were produced.

The rapid growth of juvenile *R*. *taylori* is relatively unique and alternative methods to improve model fitting could be explored beyond the scope of this study. The information theoretic approach has a limited capacity to include variations in individual growth since only a single value of L_0_ was used. Bayesian modelling on the other hand is less sensitive to missing data and can account for variation in individual growth [51, 52]. Bayesian frameworks have been used to set informative priors of L_0_ rather than fixing a specific value [51, 53]. Alternatively, since early growth of *R*. *taylori* is somewhat linear but levels off after maturity a biphasic Lester model could be suitable as a surrogate approach to approximate growth parameters in the different stages of growth [54].

The maturity estimates for *R*. *taylori* in the GOP showed that males matured within 6 months of birth while females reached maturity as they approached 12 months of age. The only other age-at-maturity estimates for *R*. *taylori* were observed by [8] and although the female age-at-maturity observed in the GOP corresponds to this study, the males in the GOP appear to reach maturity within half the time noted in Queensland. Length-at-maturity estimates for the GOP showed that males also matured at smaller sizes then females. The length at which both 50% of males and females in the GOP reached maturity resembled data from north and western Australia recorded by [15] which were smaller than that observed by [8] and [9]. These findings highlight latitudinal variation for this species suggesting length-at-maturity increases with higher latitudes. The underlying reasons for latitudinal variation in life history traits have been attributed to differences in water temperature [18, 55].

It is important to correctly determine age in sharks as errors can lead to inaccurate projections of parameters such as age-at-maturity which can have a sizable impact on population models [49], and stock assessments. Achieving accuracy and precision in vertebral aging relies on the clarity of growth markings and the ability of the readers to identify and differentiate growth bands. Several studies focused on small shark species have noted difficulties in detecting the correct number of growth bands particularly on the edge of the vertebrae, where bands are deposited very close to each other and as a consequence maximum age may be underestimated [3, 49]. Furthermore as temperate seasonality may influence the deposition of growth bands [5], they appear more pronounced in temperate sharks as opposed to tropical sharks where seasonality is limited. For instance the appearance of check marks in the GOP vertebrae were not as pronounced as that observed by [8].

Assumptions on annual growth band deposition for *R*. *taylori* were made in this study because validation was not possible due to logistic constraints. The annual periodicity of band formation for *R*. *taylori* in northern Queensland was verified by [8] based on marginal increment analysis and length frequency data. This assumption has strong support given the geographic proximity of this study, and annual band formation being the typical pattern observed in carcharhinid sharks [56, 57].

Partial ages were calculated to improve the estimation of age and overall growth model projections. This method is suited to sharks with seasonal patterns of reproduction where mating and parturition occur at specific times of the year, rather than asynchronous species. *Rhizoprionodon taylori* undergoes a seven month period of diapause where embryonic development at the blastodermic disc stage is suspended [10]. Regardless of this, the reproductive cycle of *R*. *taylori* appears to be seasonal as mating occurs only once a year from late January to early February [10]. The fertilised eggs then enter a state of diapause until September, after which active growth of the embryos recommences until parturition in January [10, 11].

The rapid growth and early onset of maturity in small-bodied sharks has been hypothesised to be a survival strategy to counter high levels of predation experienced by a species [58]. Small bodied sharks are an important intermediate link in the food chain as they are often preyed upon by larger predators [59]. A study on *R*. *acutus* by [4] also noted that high natural mortality experienced by a species may be balanced by early maturity. Certainly in the GOP *R*. *taylori* may experience high natural mortality as their small size and slower swimming capacity would render them a common prey for larger predators [58]. Furthermore, the high level of bycatch from the Gulf of Papua trawl fishery [17] places some level of fishing mortality on the *R*. *taylori* population. High levels of natural and fishing mortality may account for their very young age-at-maturity.

Commercial trawling has taken place in the GOP for over forty years. At the onset of this fishery, as many as 30 vessels were licensed. The total number of vessels and fishing effort has fluctuated over the years peaking at 95 000 trawl hours in 1989 before decreasing when effort control measures were introduced [60]. Currently only six vessels are actively trawling in the GOP. Rapid growth and early maturity are biological characteristics associated with the ability of a species to withstand fishing pressure [2], therefore it is probable that *R*. *taylori* in the GOP are better placed to withstand current fishing levels than other shark species.

The foundations of managing fish stocks and attaining sustainable fisheries rely upon accurate biological data of fish populations [25, 61]. Until recently, information for sharks in PNG has been scarce [33, 62–64]. This study is one of the first attempts to determine biological parameters of a small-bodied, commonly caught carcharhinid species in PNG. However, further work is needed to provide critical biological data for population assessments as well as to understand the ecological functions of shark species in order to fine tune management and conservation measures to suit the PNG context. Advancement in elasmobranch research in PNG will also address important data gaps for the Indo-Australasian region which supports the highest diversity of sharks globally [65].
